# Anaeromyces corallioides, sp. nov., a new anaerobic gut fungus from the faeces of cattle

**DOI:** 10.1099/ijsem.0.006719

**Published:** 2025-03-14

**Authors:** Carrie J. Pratt, Emma E. England, Julia M. Vinzelj, Noha H. Youssef, Mostafa S. Elshahed

**Affiliations:** 1 Department of Microbiology and Molecular Genetics Oklahoma State University, Stillwater, OK, USA

**Keywords:** fungi, herbivory, *Neocallimastigomycota*

## Abstract

We report on the isolation and characterization of three isolates of anaerobic gut fungi from a cattle faecal sample obtained in Stillwater, OK, USA. The isolates produced polycentric thalli with nucleated rhizomycelia, lobed appressorium-like structures, intercalary sporangia and constricted sausage-like hyphae. These morphological features are characteristic of members of the genus *Anaeromyces*. No zoospore production was observed during the isolation process or thereafter. The strains seemed to have propagated solely through their nucleated hyphae post initial enrichment. Phylogenetic analysis of the D1/D2 region of the large ribosomal subunit (D1/D2 LSU) rRNA, the ribosomal intergenic spacer region 1 (ITS1), RNA polymerase II large subunit (RPB1) and comparative average amino acid identity using transcriptomic datasets further confirmed the position of the type strain as a distinct member of the genus *Anaeromyces*, family *Anaeromycetaceae* and phylum *Neocallimastigomycota*. We propose to accommodate these isolates into a new species (*Anaeromyces corallioides*) within the genus *Anaeromyces*. The type strain is EE.1.

## Introduction

Anaerobic gut fungi (AGF) (*Neocallimastigomycota*) reside in the digestive tracts of herbivores where they aid in breaking down complex plant polysaccharides. To date, 22 AGF genera from a variety of herbivorous hosts have been described [1,2]. In addition, culture-independent surveys of rumen and faecal samples indicate the existence of many yet-uncultured genus- and family-level lineages within the *Neocallimastigomycota* [3,4].

Livestock mammals like cattle, horses, goats and sheep have traditionally been the source of most AGF isolates [5,15]. These hosts frequently harbour a highly diverse AGF community, with many AGF taxa co-occurring within a single rumen or faecal sample [4,16,20]. The AGF community within individual animals belonging to the same host species, e.g. cattle, often varies widely [3,4, 21]. Given this variation, isolation and characterization of AGF from domesticated livestock hosts will likely continue to yield novel AGF taxa despite those hosts being already well-sampled.

Further, while the number of reported AGF genera has steadily increased in the last decade [2,13, 15, 22,27], only seven genera (*Anaeromyces*, *Caecomyces*, *Capellomyces*, *Neocallimastix*, *Orpinomyces* and *Piromyces*) encompass more than one validly described species [3,4, 21]. Describing new species within known genera will enhance the taxonomic classification of AGF, illuminate gene flow and fine-scale evolutionary shifts within genera and help map the enzymatic and metabolic diversity of *Neocallimastigomycota*.

Here, as part of our continuing efforts to isolate and characterize novel AGF taxa, we report on the isolation and characterization of novel AGF isolates from a cattle herd composite faecal sample from Stillwater, OK, USA. Both morphological features and phylogenetic analysis suggest the isolates’ affiliation with members of the genus *Anaeromyces* and family *Anaeromycetaceae*, and we propose a novel species (*Anaeromyces corallioides*, sp. nov.) for their accommodation.

The family *Anaeromycetaceae* currently comprises the genera *Anaeromyces*, *Capellomyces*, *Liebetanzomyces* and *Oontomyces*, all of which are obligate anaerobic gut fungi with filamentous thallus development producing monoflagellated zoospores [28]. The clade is circumscribed by phylogenomic analysis and average amino acid identity (AAI) values and confirmed by LSU and RPB1 phylogenetic analyses. The genus *Anaeromyces* is the only one in the family displaying polycentric filamentous thalli (i.e. nucleated hyphae). Besides the novel species described in this study, the genus *Anaeromyces* currently accommodates three other validly species: *Anaeromyces mucronatus* [7], *Anaeromyces elegans* [8] and *Anaeromyces contortus* [14].

## Methods

### Samples

Faecal material (as composite samples) was collected from a captive herd of Black Angus and Hereford cattle (*Bos taurus*) located in Stillwater, OK, USA, in October 2020. Composite samples, taken from a heap that contained deposits from multiple individuals, were collected in 50-ml conical centrifuge tubes and transferred on ice to the laboratory within 24 h of collection. Upon arrival, samples were stored at −20 °C.

### Isolation

Enrichments were set up in an anaerobic chamber (Coy Laboratories, Grass Lake, MI, USA) in August 2023 and July 2024 by diluting thawed faecal material (1 g) into rumen fluid cellobiose (RFC) media [29] supplemented with antibiotics (50 µg ml^−1^ chloramphenicol, 20 µg ml^−1^ streptomycin and 50 µg ml^−1^ penicillin) and switchgrass (0.1 g ml^−1^). The enrichments were incubated stationary at 39 °C. Enrichments with clumping and floating plant material, visible fungal biomass and/or gas bubbles were subcultured into fresh media containing no switchgrass, and 1 ml was rolled on RFC agar roll tubes [30,31]. Roll tubes were incubated until colonies were visible, and single colonies were picked into liquid RFC media. The process was repeated twice before the strain was identified by Sanger sequencing of the D1/D2 region of the LSU. Strains are maintained by biweekly subculturing into RFC media and incubation at 39 °C.

### Morphological characterization

Growth patterns and colony morphology were observed in liquid RFC media and roll tubes. Light microscopy was performed on an Olympus BX51 microscope equipped with a DP71 digital camera. Confocal microscopy was performed on a Zeiss LSM 980 Airyscan 2 (Carl Zeiss AG) confocal laser scanning microscope to examine nuclei localization in samples stained with 4,6′-diamidino-2-phenylindole (10 µg ml^−1^) as previously described [13]. Scanning electron microscopy (SEM) was performed on a FEI Quanta 600 field-emission gun environmental scanning electron microscope with a Bruker EDS X-ray microanalysis system and an HKL EBSD system. For SEM, samples were fixed and critical point dried as previously described [13].

In an attempt to identify zoospores and terminal sporangia, the isolate was transferred to a modified sloppy medium with agar and cellulose (0.1% w/v) to stimulate their production as previously described [32]. The isolates were further starved on roll tubes for 2 weeks before being transferred into liquid RFC media containing switchgrass. The potential production of sporangia and zoospores was monitored daily using light microscopy. Neither method led to the observation of terminal sporangia or zoospores.

### Substrate preferences

**Fig. 1.**
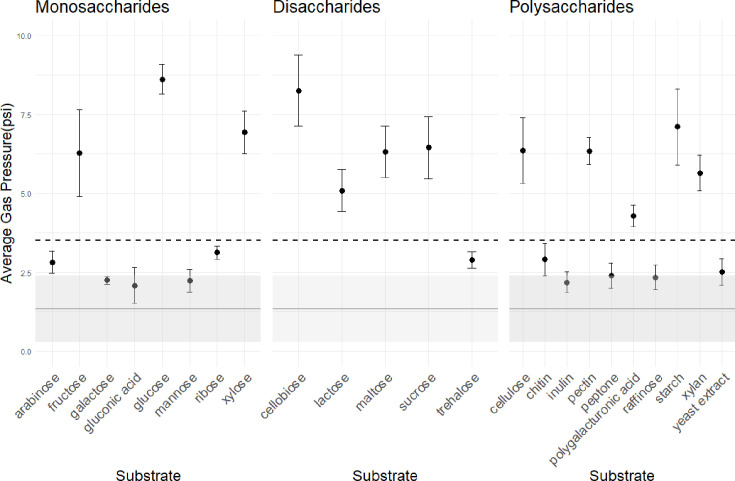
Substrate utilization. Gas pressure (PSI) values obtained when growing *A. corallioides* cultures on 23 different substrates. Gas pressure measurements are used as a proxy for the growth of anaerobic gut fungi. For each substrate, *A. corallioides* was grown in four replicates over three consecutive subcultures (each incubated for 5 days at 39 °C). Substrate-unamended media and uninoculated tubes served as negative controls. The gas pressure change (between day 1 and day 5) was averaged across all replicates and subcultures per substrate (*n*=12 for each substrate and *n*=24 for the negative controls) and is shown here together with error bars indicating the standard deviation. The respective average of the negative controls is shown as a dark grey horizontal line, with the lighter grey areas indicating the standard deviation. Qualitative visual evaluation of growth was recorded together with PSI values. It correlated very well with the PSI values (tubes with lower gas pressure also showed less biomass production). The black dotted line represents the combined results of visual inspection and gas pressure measurements: substrates with gas pressure values below the dotted line did not support the growth of *A. corallioides* across three consecutive subcultures, and substrates with gas pressure values above that line did. Substrates were deemed supportive of growth if all three consecutive subcultures showed growth of *A. corallioides*.

The type strain was grown on 23 different substrates (0.5% w/v) with antibiotics (50 µg ml^−1^ chloramphenicol, 20 µg ml^−1^ streptomycin and 50 µg ml^−1^ penicillin) in rumen fluid (RF) media without cellobiose. Substrates included monomers (arabinose, fructose, galactose, gluconic acid, glucose, mannose, ribose and xylose), dimers (cellobiose, lactose, maltose, sucrose and trehalose), polymers (cellulose, chitin, inulin, pectin, peptone, polygalacturonic acid, raffinose, starch and xylan) and yeast extract. Given the large chemical variability in polymers, the sources for each polymer are as follows: Sigmacell Cellulose Type 50 (Sigma-Aldrich), chitin powder (Alfa Aesar), inulin (Spectrum Chemical Corporation), pectin from citrus (TCI Corporation), polygalacturonic acid (Alfa Aesar), acid casein peptone (Fisher BioReagents), d-(+)-raffinose pentahydrate (Alfa Aesar), Difco™ Soluble Starch (BD), xylan from beechwood (Sigma) and yeast extract (Thermo Scientific). RF substrate-unamended media and uninoculated RF media served as negative controls. Four replicates were made for each substrate. All cultures underwent three consecutive subcultures, each lasting 5 days. Growth was assessed both visually (biomass production, biomass sticking to glass) and by measuring headspace accumulated gas pressure using a digital pressure gauge (MediaGauge, SSI Technologies). Pressure was measured at the start and end of incubation periods immediately prior to subculturing, and the difference between those start and end values per replicate and 5-day growth period was used to calculate the average pounds per square inch (PSI) change for each substrate. Negative changes in pressure were attributed to faulty Balch tube stoppers, and values were discarded before averaging replicates. R (version 4.4.1) and the R packages tidyverse (version 2.0.0) [33], patchwork (version 1.3.0) [34] and ggplot2 (version 3.5.1) [35] were used to plot the results in Fig. 1.

### Phylogenetic analysis and ecological distribution

Cultures were vacuum filtered to obtain fungal biomass. DNA extraction was conducted using a DNeasy PowerPlant Pro Kit (Qiagen Corp., Germantown, MD, USA) according to the manufacturer’s instructions. The D1/D2 domains of the 28 S rRNA gene were amplified using the primers NL1 (5′-GCATATCAATAAGCGGAGGAAAAG-3′) and NL4 (5′-GGTCCGTGTTTCAAGACGG-3′), cleaned using a PureLink PCR Purification Kit (Thermo Fisher Scientific, Waltham, MA, USA) and Sanger-sequenced. For the type strain EE.1, the entire region encompassing ITS1-5.8S rRNA-ITS2-D1/D2 domains of the LSU rRNA gene was amplified using the primers ITS5 (5′-GGAAGTAAAAGTCGTAACAAGG-3′) and NL4 and cloned into a pCR-XL-2-TOPO cloning vector according to the manufacturer’s instructions (Thermo Fisher Scientific). Sixteen clones were Sanger-sequenced at the Oklahoma State University DNA Protein Core Facility (Stillwater, OK, USA). Regions corresponding to the ITS1 and D1/D2 LSU were extracted in mega [36]. In addition, and as previously suggested [28], we used the RPB1 as an additional marker for taxonomy. The amino acid sequence of RPB1 was obtained from the *Anaeromyces robustus* genome (GenBank assembly accession number: GCA_002104895.1) and used as bait for blastp searches against strain EE.1 predicted proteomes from the strain’s assembled transcriptomic dataset (see below).

Strain EE.1 RPB1 predicted protein sequence, and rRNA loci were aligned to reference sequences using MAFFT [37] with default parameters. The alignments were then fed into IQ-TREE [38,41], first to predict the best substitution model and then to construct maximum likelihood trees under the predicted best model. The IQ-TREE command line included the ‘–alrt 1000’ option for performing the Shimodaira–Hasegawa approximate likelihood-ratio test (SH-aLRT), the ‘-abayes’ option for performing approximate Bayes tests (aBayes) and the ‘–bb 1000’ option for ultrafast bootstrap (UFB). Generated phylogenetic trees thus included three support values (SH-aLRT, aBayes and UFB) on each branch.

We conducted blast searches using the D1/D2 region of strain EE.1 against several AGF culture-independent datasets [3,4, 42, 43] amplifying the D2 region of the LSU rRNA. We considered 99% similarity as the cutoff to define hits.

### Transcriptome sequencing and AAI calculation

Strain EE.1 was grown in an RFC medium for 4 days, followed by vacuum filtration and fungal biomass grinding under liquid nitrogen. Epicentre MasterPure Yeast RNA Purification Kit (Epicentre, Madison, WI) was used for total RNA extraction according to the manufacturer’s instructions. Transcriptomic sequencing was conducted on the Illumina NextSeq 1000/2000 platform and a 2×150 bp paired-end library using the services of the Oklahoma State University Genomics and Proteomics Center. The RNA-seq data were quality-trimmed and *de novo* assembled with Trinity (v2.6.6) using default parameters [44]. The obtained transcripts were subsequently used for peptide and coding sequence prediction using TransDecoder (v5.0.2) [45] with a minimum peptide length of 100 amino acids. We calculated AAI values using the aai.rb script available as part of the enveomics collection [46] to compare the EE.1 predicted proteome dataset to all previously available *Anaeromyces* proteome datasets (*n*=6), as well as predicted proteomes from other genera in the *Anaeromycetaceae* family [28]. These included one *Liebetanzomyces* and two *Capellomyces* datasets.

### Data and culture accession

Sequences generated in this study are deposited in GenBank under the BioProject accession numbers PRJNA1169334. The RNA-seq read data are available under BioSample accession number SAMN44072218. Clone sequences of the region encompassing ITS1-5.8S rRNA-ITS2-D1/D2 domains of the LSU rRNA gene are available under accession numbers PQ464100 to PQ464115. Cultures are available at the Oklahoma State University, Department of Microbiology and Molecular Genetics culture collection (Stillwater, OK, USA). Cultures are maintained as active cultures (RFC medium, bi-weekly subcultivation, incubation stationary at 39 °C) and preserved and stored at −80 °C (protocol CP_eg_) [47].

## Results

### Isolation

Three strains (EE.1, EE.2 and EA.1) were isolated from the cattle herd’s faecal sample. All strains displayed identical colony morphology, microscopic features and D1/D2 LSU sequences. Strain EE.1 was selected for detailed characterization and designated as the type strain.

### Morphological characterization

**Fig. 2.**
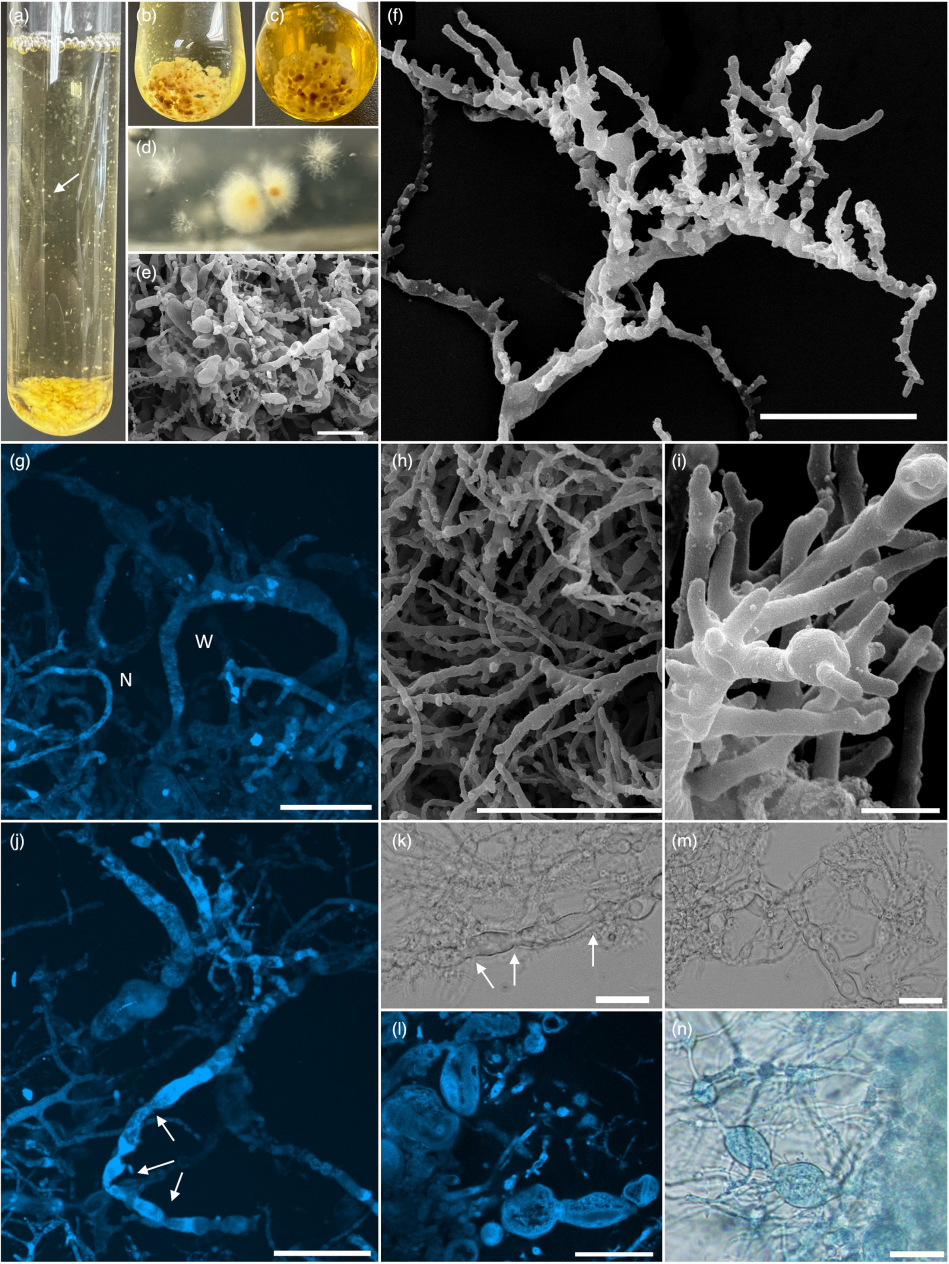
Macroscopic and microscopic features of *A. corallioides* strain EE.1, observed using light (k, m, n), confocal (g, j, l) and scanning electron microscopy (e, f, h, i), and pictures showing growth in liquid (a–c) and solid (d) media. Polycentric thallus development with nucleated rhizomycelia (g, j, l). Both wide (w) and narrow (n) hyphae were observed (2G). The *Anaeromyces*-typical trait of constricted sausage-like morphology (arrows in j, k). Hyphae were highly branched, typically with short, knobby branches (f, h). Lobed appressorium-like structures were observed (I) in addition to intercalary sporangia (e, l, n). Scale bars indicate 20 µm (e, f, g, h, j, k, l, m, n) and 2 µm (I).

In liquid RFC media, strain EE.1 formed dense pearl-like granules that increased in size and darkened in colour from white to dark brown with culture age (Fig. 2a–c). With time, granules would clump together (Fig. 2b, c), and on occasion, minute granules would affix to the sides of the Balch tube (Fig. 2a). On agar roll tubes, strain EE.1 formed small white colonies with visible filamentous hyphae and a dense centre that darkened with culture age (Fig. 2d).

Strain EE.1 displayed a polycentric thallus development pattern with nucleated rhizomycelia (Fig. 2g, j and l). Both wide (W) and narrow (N) hyphae were observed (Fig. 2g). Wide hyphae varied, with some being entirely smooth and not constricted, and others displaying the *Anaeromyces*-typical trait of constricted sausage-like morphology (Fig. 2j, k). Thin hyphae were highly branched, typically with short, knobby branches (Fig. 2f, h1). Branches did not appear to be evenly distributed along hyphae and varied in proximity to one another. Lobed appressorium-like structures similar to those described in other *Anaeromyces* species [14] were infrequently observed (Fig. 2i).

Strain EE.1 produced intercalary sporangia, i.e. sporangia that develop as lateral outgrowths of the hyphae or from hyphal expansion (Fig. 2e, k, l and n). These hyphal expansions were observed at irregular intervals along hyphae and varied in size, as did the constrictions between them. No clearly defined terminal sporangia were observed in our culture, although it is likely that the isolate rapidly lost the ability to produce sporangia and zoospores after initial enrichment, probably developing new biomass through hyphal propagation as characteristic with polycentric filamentous AGF like members of the genus *Anaeromyces* [1,12].

### Phylogenetic analysis

**Fig. 3.**
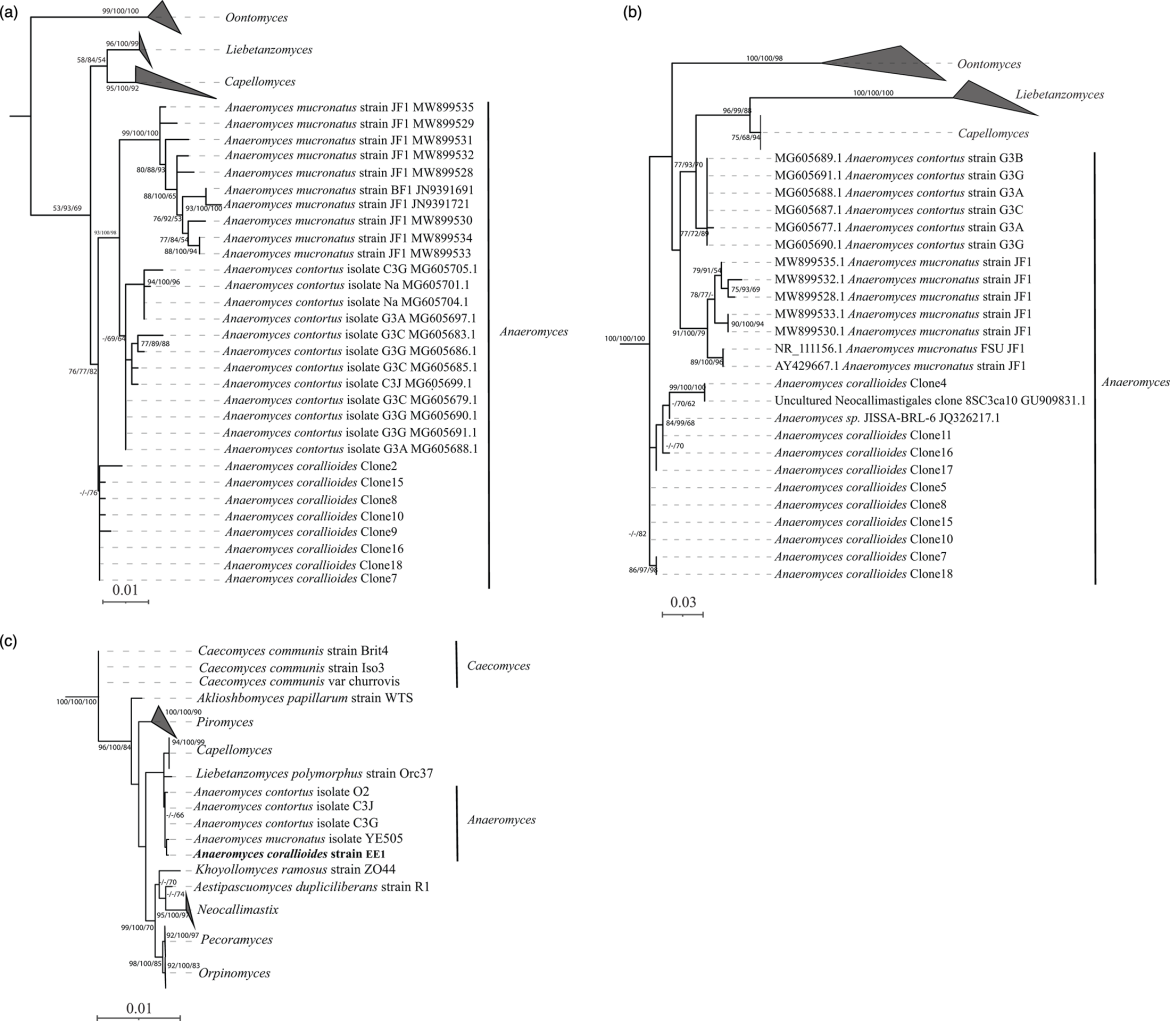
Assessment of the phylogenetic position of EE.1 using D1/D2 LSU (a), ITS1 (b) and RPB1 (c). The RPB1-predicted protein sequence for RPB1 and rRNA loci D1/D2 and ITS1 of EE.1 were aligned to reference sequences using MAFFT with default parameters. The alignments were then fed into IQ-TREE, first to predict the best substitution model and then to construct maximum likelihood trees under the predicted best model. Through IQ-TREE, the SH-aLRT, aBayes and UFB were performed. Therefore, generated phylogenetic trees include three support values (SH-aLRT, aBayes and UFB) on each branch. Support values >50 are shown. To avoid cluttering the tree, not all 16 clones for *A. corallioides* are shown. Identical clones were merged.

Sixteen clones covering the full region extending from ITS1 to the end of the D2 region of the LSU rRNA were Sanger-sequenced. Phylogenetic analysis using the D1/D2 region placed the clones as a distinct clade within the genus *Anaeromyces* (family *Anaeromycetaceae*) that diverged 2.23–4.63% (ITS1; average 2.63%) and 0.98–1.62% (D1/D2; average 1.12%) from *A. contortus* clones and 2.51–4.22% (ITS1; average 3.1%) and 1.72–2.93% (D1/D2; average 2.24%) from *A. mucronatus* clones (Fig. 3a, b). Within strain divergence ranged between 0–2.22% (ITS1; average 0.89%) and 0–0.37% (D1/D2 region; average 0.14%). These thresholds are within the recommended criteria for assigning new species [28,48]. Phylogenetic analysis using RPB1 placed strain EE.1 within the genus *Anaeromyces* (% similarity values 99.76±0.28%) and within the *Anaeromycetaceae* family with 98.78± 0.38% similarity to the type species of the genus *Capellomyces* and 97.22% similarity to the type species of the genus *Liebetanzomyces* (Fig. 3c).

### Ecological distribution

*A. corallioides* showed a limited ecological distribution. The comparison of the ITS1 sequences from the 16 EE.1 clones to the nr database (at >96% similarity) identified only seven sequences (coming from one isolate JQ326217.1; an *Anaeromyces* species isolated from a Murrah buffalo in India) and six uncultured clones (GU909849.1, GU909887.1, GU910222.1, GU910233.1, GU910236.1 and GU910249.1), coming from domesticated cow faeces in New Mexico [49]. Based on inter-species divergence to *A. contortus* and *A. mucronatus*, these seven sequences could potentially also belong to the *A. corallioides* clade.

Using the LSU rRNA sequences of strain EE.1, we conducted local Blast searches against recent AGF culture-independent datasets that amplified the D2 domain of the LSU rRNA [3,4, 42, 43]. Using 99% as the similarity cutoff (based on the narrow inter-species LSU divergence values to *A. contortus*), *A. corallioides* was only encountered in a wallaby (1 sequence) [43] and 18 cows (a total of 124 sequences), 2 deer (2 sequences), 2 goats (a total of 7 sequences), 1 horse (1 sequence) and 1 sheep (1 sequence) [4]. No *A. corallioides* sequences were encountered in tortoises [42] or in a recent study by Young *et al*. [3]. In all samples where * A. corallioides* was identified, its percentage abundance never exceeded 0.063% of the total AGF community.

### Substrate preferences

Substrate preferences are shown in Fig. 1. Growth of strain EE.1 was supported by the monomers fructose, glucose and xylose; the dimers cellobiose, lactose, maltose and sucrose; and the polymers cellulose, pectin, polygalacturonic acid, starch and xylan. However, growth on polygalacturonic acid was weak. Substrates not supporting growth included monomers arabinose, galactose, gluconic acid, mannose and ribose; dimer trehalose; polymers chitin and inulin; peptone; raffinose; and yeast extract.

### Average amino acid identity

Strain EE.1 showed high AAI values compared to all other *Anaeromyces* strains (93.15±0.32% for *A. contortus* and 95.31% for * A. mucronatus*) and slightly lower AAI values with other members of the *Anaeromycetaceae* family (85.16±0.52% with *Capellomyces* species and 84.43% with *Liebetanzomyces* species, confirming its position in the genus and the clade.

## Discussion

The genus *Anaeromyces* is described as having a polycentric rhizoidal developmental pattern, filamentous hyphae with sausage-like constrictions and uniflagellated zoospores [1]. Three species of *Anaeromyces* have been described with these defining characteristics: *A. mucronatus* [7], *A. elegans* [8] and *A. contortus* [14]. Both *A. mucronatus* and *A. elegans* are highly similar in morphology. The lack of type strains and sequence data from both species hinders further confirmation of synonymy, as previously noted [1]. A fourth species that has previously been described is *A. robustus* [12]. However, this species differs from the other *Anaeromyces* species in that it produces club-shaped sporangia and lacks the *Anaeromyces*-specific trait of hyphae with a constricted, sausage-like morphology. The most recent review of anaerobic gut fungi taxonomy indicates that *A. robustus* likely belongs to the genus *Capellomyces* [1].

Microscopic characteristics (Fig. 2) support the placement of strain EE.1 as a member of the genus *Anaeromyces*. However, strain EE.1 appears unique in having very highly branching hyphae (Fig. 2f) compared to the less frequently branching hyphae of *A. mucronatus* and *A. contortus*. Whether this is a function of sample preparation, differences in media composition or a true reflection of hyphal growth patterns in its natural environment is currently unclear.

Phylogenetically, strain EE.1 exhibited 1.12±0.18 % and 2.24±0.23% sequence divergence in D1/D2 LSU from *A. contortus* and * A. mucronatus*, respectively, and 2.63±0.56 % and 3.09±0.48% sequence divergence in the ITS1 region. This within strain variation in the ITS1 region is similar to the dissimilarity values observed for *A. contortus* and *A. mucronatus* (2.91±0.49%) and is within the recommended cutoff for describing new species [1]. AAI values from whole transcriptome analysis also showed 93.15% similarity to *A. contortus* and 95.31% similarity to *A. mucronatus*. Such values are within the circumscribed boundaries proposed for describing new species within the *Neocallimastigomycota* (between 87 and 99%) [28].

Within the family of *Anaeromycetaceae*, substrate utilization data is only available for the type strain of *Liebetanzomyces* and one of the three validly described *Anaeromyces* species (*A. mucronatus*) [7]. All three strains, *Liebetanzomyces polymorphus*, * A. mucronatus* and *A. corallioides*, can grow on cellobiose, fructose, glucose, maltose, sucrose and xylose, but not on arabinose, galactose, mannose or raffinose. Lactose supported only weak growth for *L. polymorphus* and *A. mucronatus*, while the growth of *A. corallioides* on lactose was stable. The polymers cellulose, starch and xylan were supporting the growth of all strains again, while pectin supported only *L. polymorphus* and *A. corallioides*. Only *A. corallioides* was able to grow on polygalacturonic acid, albeit weakly. Polymers, however, can be highly variable in their chemical structure and hence their degradation requirements, making comparisons like this difficult if the sources are unknown.

The genus *Anaeromyces* belongs to the family *Anaeromycetaceae*, which also encompasses the genera *Capellomyces*, *Oontomyces* and *Liebetanzomyces* [28]. Interestingly, inter-genus sequence variability in the D1/D2 LSU rRNA gene, the ITS1 region, the RPB1 protein sequence and AAI pairwise values in the *Anaeromycetaceae* are narrower when compared to other families in the *Neocallimastigomycota* [28]. However, the fact that the genus *Anaeromyces* is defined by polycentric rhizoidal growth patterns while all other genera in the family *Anaeromycetaceae* are monocentric justified the accommodation of these monocentric strains as genera distinct from *Anaeromyces*. In this study, the EE.1 strain displayed sequence divergence values in line with accommodating it as a new species using the broader criteria used for the phylum *Neocallimastigomycota*. The sequence divergence is also within the range of inter-genus diversity values in the family *Anaeromycetaceae*. However, while other genera in the family display cardinal microscopic differences from the genus *Anaeromyces*, strain EE.1 displays the distinctive morphological and microscopic characteristics of members of the genus *Anaeromyces* (polycentric growth pattern, pearl-like granular growth in liquid media and formation of constricted, sausage-like hyphae (Fig. 2) as stated above). As such, until further evidence suggests otherwise, we reason that strain EE.1 should be described as a novel species in the genus *Anaeromyces* rather than a novel genus in the family *Anaeromycetaceae*.

In summary, we here described and characterized a novel species of AGF isolated from a composite cattle faecal sample. This species was rarely (relative abundance <0.063 %) observed in other animals (based on recent LSU amplicon sequencing studies) [3,4, 42, 43]. Obtaining a novel AGF taxon from arguably the most sampled host animal demonstrates that isolating novel uncultured species – and perhaps genera – from well-sampled animal hosts is still possible. Phylogenetic affiliation, microscopic features and phenotypic characteristics justify proposing a new species within the genus *Anaeromyces* to accommodate these isolates, for which the name *A. corallioides* is proposed.

## Description of *Anaeromyces corallioides* sp. nov.

CJ Pratt, EE England, JM Vinzelj, NH Youssef, and MS Elshahed.

*Anaeromyces corallioides*: co.ral.li.o’i.des. Gr. neut. n. korallion, coral; L. adj. suff. –oides (from Gr. neut. adj. suff. –eides, from Gr. neut. n. eîdos, that which is seen, form, shape, figure), resembling, similar; N.L. masc. adj. corallioides, coral-shaped).

Isolated in August 2023 from the frozen, then thawed faeces of a herd of cattle (composite sample) located in Stillwater (USA, Oklahoma, Stillwater). A metabolically active ex-type strain EE.1 is maintained under anaerobic conditions by biweekly subculture at 39 °C. The holotype is stored at the Oklahoma State University in a 4% glutaraldehyde solution. DNA from the ex-type strain is stored at −80 °C at the Oklahoma State University. GenBank accession numbers PQ464100 to PQ464115 (the region encompassing ITS1-5.8S rRNA-ITS2-D1/D2 domains of the LSU rRNA gene).

An anaerobic fungus with a polycentric thallus development pattern. Produces wide and narrow nucleated hyphae. Wide hyphae are characterized by constricted sausage-like morphology. Narrow hyphae are characterized by extensive branching and short, blunted rhizoids. Sporangia may be intercalary and globose. Granular growth in cellobiose-containing liquid media and small white colonies on agar roll tubes. MycoBank ID for the species is MB 856119

Additional specimens examined: Two additional isolates (EE.2 and EA.1) belonging to *A. corallioides* were isolated from the same composite sample in 2023 and 2024.

## Abbreviations

AAI: average amino acid identity
aBayes: approximate Bayes tests
AGF: anaerobic gut fungi
RF: rumen fluid
RFC: rumen fluid cellobiose
SEM: scanning electron microscopy
SH-aLRT: Shimodaira–Hasegawa approximate likelihood-ratio test
UFB: ultrafast bootstrap
